# Calcaneus Fracture Fixation in Prone Position: A Novel Technique and Case Series

**DOI:** 10.7759/cureus.28480

**Published:** 2022-08-27

**Authors:** Khalid Hasan, Mazin Foodoul, Shahin Kayum, Hasan AbuHejleh, Mohammed AlKhayarin

**Affiliations:** 1 Orthopaedic Surgery, Virginia Commonwealth University School of Medicine, Richmond, USA; 2 Orthopaedic Surgery, Hamad General Hospital, Doha, QAT; 3 Orthopaedic Surgery, University of Toronto, Toronto, CAN

**Keywords:** open reduction internal fixation, complications, technique, prone position, calcaneus fractures

## Abstract

Introduction: Calcaneus is one of the most commonly fractured tarsal bones. The use of computed tomography (CT) has enabled more accurate fracture configuration and classification of fractures. The outcomes of operative versus nonoperative treatment of these fractures have been extensively debated with variable results. Significant complications following intra-articular fractures have been reported in the literature despite management by experienced surgeons. This article will discuss the treatment of calcaneus fractures by open reduction and internal fixation in a prone position with a calcaneal traction pin, and assess the outcomes following this novel technique.

Methods: Fifty-three patients with 58 acute displaced intra-articular calcaneal closed fractures (Sanders type II and III), presented to one tertiary center, were treated by open reduction and internal fixation in a distracted prone position through an extensile lateral approach by a single surgeon and assessed for postoperative wound and soft tissue complications. The primary outcome measure was postoperative wound complications. Secondary outcome measures were postoperative ankle and subtalar range of motion and return to work. Radiological assessment of anatomical reduction by measuring preoperative and postoperative Bohler’s angle, Gissane angle, and posterior facet joint depression was conducted. A comparison was made with paired sample t-test with a confidence interval of 95%.

Results: Fifty-three patients with 58 calcaneus fractures were treated surgically. Three cases (5.6%) developed postoperative wound infection, of which only one needed surgical intervention. The reoperation rate was 5.6% in our study. Half of the patients (50%) were able to be followed up long-term, and the radiographs showed significant restoration of Bohler’s angle and posterior facet joint depression in 24 patients. Return to full duties was achieved in an average of 5.6 months for 16 of 22 patients, who were available for follow-up questions with regard to return-to-work status.

Conclusion: Operative treatment of calcaneus fracture by open reduction and internal fixation in the novel distracted prone position technique has shown a low rate of the wound and soft tissue complications and can be considered as an alternative approach in treating these fractures.

## Introduction

Calcaneus fractures make up about 2% of all fractures and calcaneus is the most frequently injured tarsal bone [1]. It is often stated that these fractures are mainly occupational injuries, which usually occur during falls from height in young males or as a result of road accidents, which frequently require complex reconstructive surgery and cause significant long-term disability [2,3].

Clinical studies have examined the effectiveness of operative versus nonoperative treatment of these fractures [4,5], and the use of computed tomography (CT) has enabled more accurate fracture configuration and classification of fractures. Significant complications following intra-articular fractures have been reported despite management by experienced surgeons [6]; although complications have followed both operative and non-operative treatment, operative treatment has been found superior to conservative management in an indicated fracture pattern [7].

Soft tissue complications, such as compartment syndrome, fracture blisters, full-thickness skin necrosis, and peroneal tendon pathology, can be seen in patients treated non-operatively, illustrating the point that even conservative management can result in suboptimal outcomes. The two most feared complications in treating calcaneus fractures with open reduction and internal fixation are wound necrosis and deep infection [8], especially with the most popularized and commonly used extensile lateral approach [9-11].

In this study, we share our experience in treating this kind of fracture through an extensile lateral approach with the patient lying in a distracted prone position. Our goal is to report our experience with this technique and study the following outcomes: soft tissue complications, return of patients to work, ankle and subtalar joints’ range of motion, and anatomical reduction of the fracture as assessed radiologically by measuring Bohler’s angle, Gissane angle, and posterior facet joint depression through pre- and postoperative radiographs. We hypothesized that this technique would allow for restoration of the calcaneus anatomy with a low wound complication.

## Materials and methods

In this retrospective case review, we reviewed 53 patients with 58 acute displaced intra-articular calcaneal closed fractures (Sanders type II and III) who were presented to a tertiary center from October 1999 to June 2012 and treated by open reduction and internal fixation in a distracted prone position through an extensile lateral approach. All 53 cases were performed by the senior surgeon using the same technique. Institutional review board (IRB) exemption and waiver of consent were obtained for the purpose of the study. The main aim of the study was to evaluate wound healing issues [12,13], restoration of radiographic parameters, and return-to-work status.

Inclusion criteria for the study included intra-articular displaced calcaneus fracture, closed, acute traumatic fracture, and Sanders classification types II and III. Patients with open fractures and fractures of Sanders classification types I and IV who were operated on with any other technique [14,15] were excluded from the study. Patients were admitted to our hospital on the same day of injury. Their fracture(s) were assessed by plain radiographs and CT scans of calcaneum (lateral and axial view) before being classified according to the classification system of Sanders et al. [16,17] based on the number and location of articular fragments alone, which they found to be useful in determining both treatment and prognosis after surgical management. Patients who met the inclusion criteria were initially managed by high elevation of the injured foot with ice pack application and non-steroidal anti-inflammatory medications. The patient was considered to be ready for surgery once the injured foot’s swelling improved with a positive wrinkle sign usually in about 10-14 days.

Operative technique

After induction of general anesthesia, a high thigh tourniquet was applied, followed by the patient being transferred to the operating room table in the prone position. Prophylactic antibiotics were given within 1 hour of the incision. Standard prepping and draping were performed under aseptic conditions; the tourniquet was inflated to 350 mm Hg. Timeout was performed and verified by all team members. A 4.5 mm Steinmann pin was inserted from medial to lateral in the calcaneus tuberosity and bent from both sides in a U-shape.

**Figure 1:**
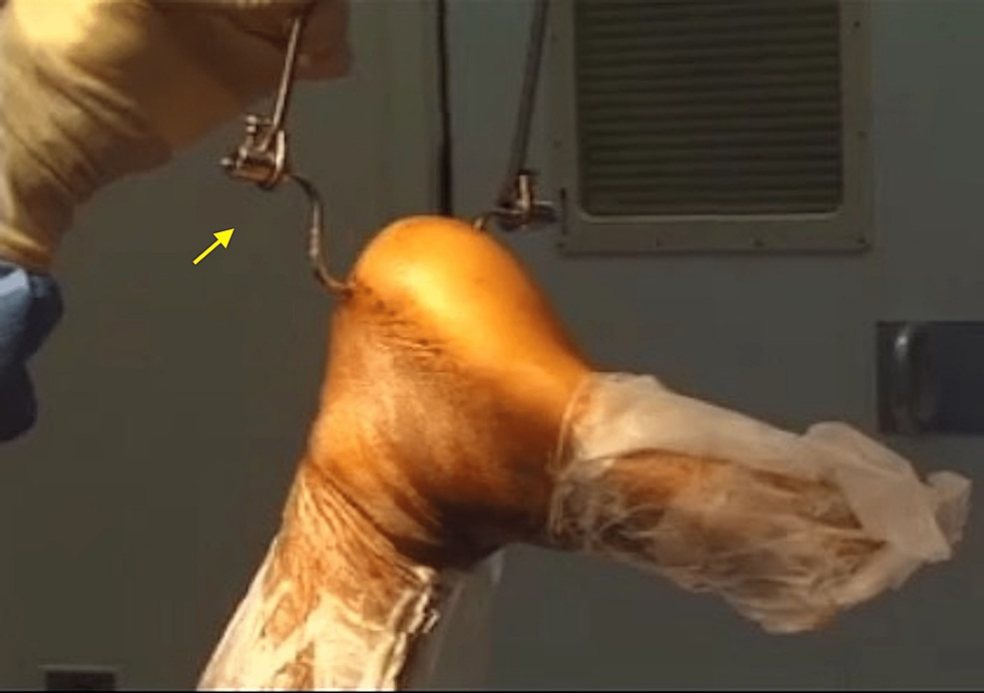
Pin traction setup Demonstration of the overhead traction by attaching a horse-shoe skeletal traction ring to the manually bent Steinmann pin

Overhead traction was applied by attaching a horse-shoe skeletal traction ring to the bent Steinmann pin and then hanging the leg by the ring to a rope hanging down from the ceiling of the operating theater. Sterile hooks were used to connect the ring to the rope (Figure 1).

The traction was adjusted to make sure the knee of the patient was kept just above the table level and not resting on the operation table, taking advantage of the distraction by gravity and using the weight of the limb to distract and reduce the fracture. The traction distracts the subtalar joint and corrects the tendency of the calcaneum to drift into varus, which can happen when the patient is in the lateral or supine position.

**Figure 2:**
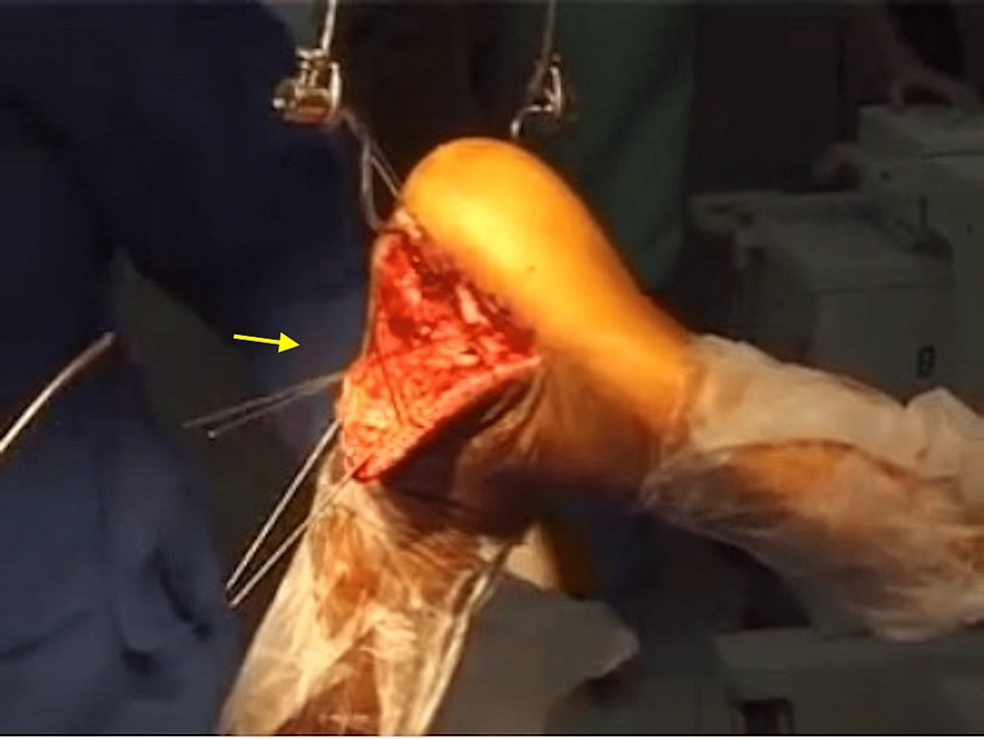
Fracture approach and flap retraction The flap retracts due to gravity and the retraction is secured using Kirschner wires

The calcaneum was then approached through an extensile lateral L-shaped incision starting just anterior to the Achilles tendon to the base of the fifth metatarsal bone. A full-thickness flap that includes the peroneal tendon and the sural nerve was developed. Then, using two thin 0.062 Kirschner wires inserted in the talus and bent proximally, the flap was held retracted, and dealt with by a “no-touch technique” throughout the procedure (Figure 2).

The fracture was approached through the lateral wall, which was hinged out like a door. Then, the posterior facet fragments were reduced and fixed using Kirschner wires. The resulting cavity after the reduction was filled with bone grafts in 42 (72%) fractures (39 with autogenous grafts obtained from the iliac crest, two with synthetic graft, and one using both). The lateral wall was then closed, and a contoured plate was applied laterally and fixed with the appropriate screws. Intraoperative lateral and axial views were taken to confirm the reduction and implant placement.

The wound was closed in two layers. First, the fascia was closed with absorbable sutures (vicryl 2/0); second, the skin was sutured using Allgower modification of the Donati technique [2] with non-absorbable sutures (Ethilon) to minimize further injury to the distal flap. A mini drain was kept, and prophylactic antibiotics were continued postoperatively for 24 hours.

Postoperative care

**Figure 3:**
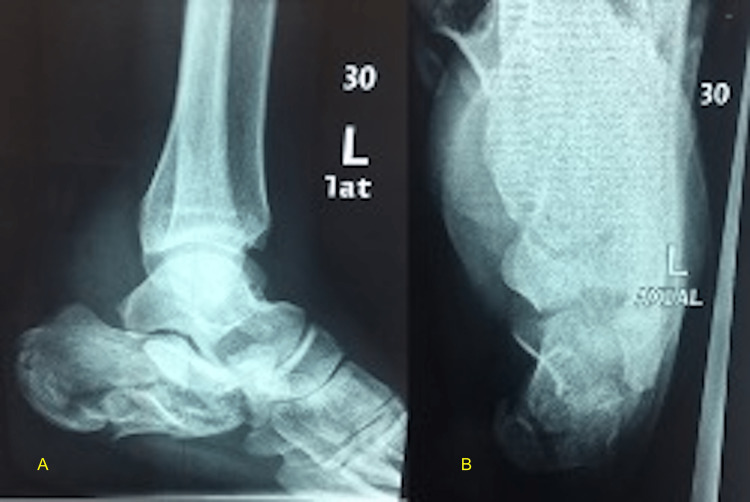
Preoperative radiographs Preoperative radiographs of calcaneus fracture of a 31-year-old gentleman that was operated using the distracted prone positioning. A) Lateral view; B) axial view

**Figure 4:**
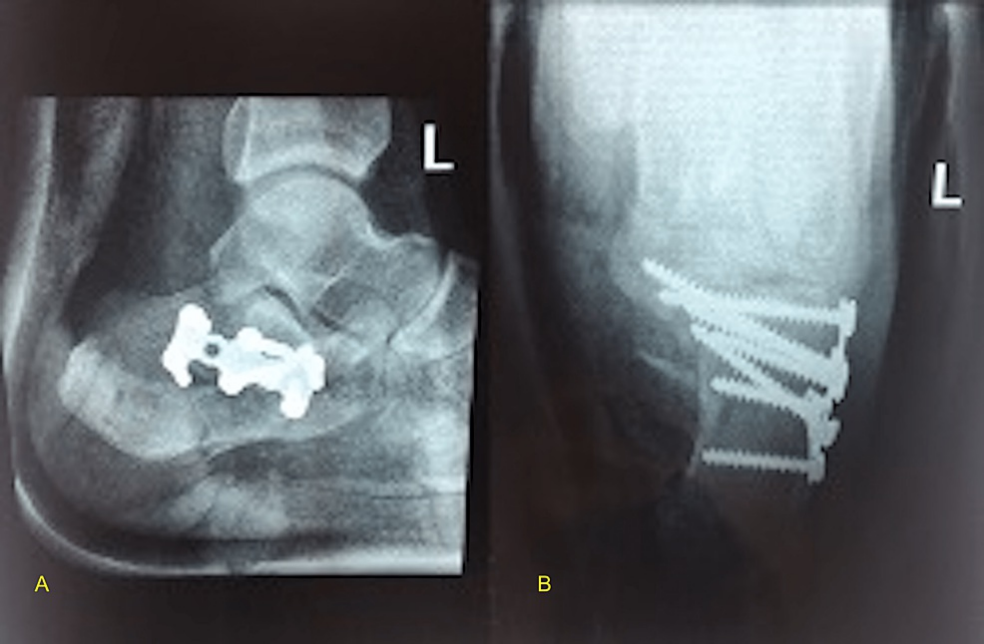
Postoperative radiographs Postoperative radiographs of the same 31-year-old gentleman after undergoing open reduction internal fixation of the right calcaneus fracture with the distracted prone setup demonstrating appropriate reduction and restoration of joint surface. A) Lateral view; B) axial view

The drain was removed after 24 hours; the wound was checked on the third and fifth postoperative days. The patient was discharged from the hospital with non-weight-bearing ambulation instructions in a posterior plaster of Paris splint. The nylon skin suture was removed after two weeks in the office visit. Radiographs were obtained at two weeks during all subsequent visits (Figures 3, 4). Eight to ten weeks postoperatively, the patients were instructed gradual weight bearing with their normal shoes, and range-of-motion exercises of the ankle and subtalar joints were initiated.

## Results

There were 58 intra-articular calcaneus fractures in 53 patients. There were 49 male (92.5%) and four female (7.5%) patients (Table 1). The average age was 34.3 years (range: 19-59). The mechanism of injury was fall from a height in 47 patients (88.7%), slip with twisting injury in five patients (9.4%), and motor vehicle collision in one patient (2%) (Table 2).

**Table 1:** Gender distribution Demographic distribution of the patients who underwent surgical fixation for calcaneus fractures in distracted prone position

Gender	Frequency	Percent
Female	4	7.5
Male	49	92.5
Total	53	100.0

**Table 2:** Mechanism of injury Tabular demonstration of the distribution of cases according to the mechanism of injury MVA, motor vehicle accident

Mechanism of injury	Frequency	Percent
Fall from height	47	88.7
Slip	5	9.4
MVA	1	1.9
Total	53	100.0

Regarding comorbidities, there were no patients who had diabetes mellitus; 46 patients had no past medical illness at the time of injury; one had hypertension and one had bronchial asthma; five had an unknown past medical history. Forty-seven patients (88.6%) were non-smokers and two patients were active smokers (3.7%). One of the two patients who smoked had a wound infection that was treated with debridement, and the second one had wound edge necrosis that was treated conservatively. There were four patients whose smoking history was unknown.

The right side was affected in 29 patients (55%) and the left side in 19 patients (36%). The bilateral injury occurred in five patients (9%). The fractures were classified according to Sanders classification - 60% were found to be of type II (OTA 82-C2) and 40% of type III (OTA 82-C3). Types I (OTA 82-C1) and IV (OTA 82-C4) were excluded from the study.

There were a total of three cases (5.5%) with wound infection and one case of a hematoma, which were managed with local measures and antibiotics. There were four cases (7.5%) of wound edge necrosis that were managed expectantly. Two wound infections were superficial and were treated with dressing and antibiotics; one case was managed by surgical debridement.

There was no radiographic evidence of subtalar arthritis during our follow-up. We had a wide range of follow-ups, which averaged 8.4 months (16 days-19 months). The nature of work after injury was assessed in 22 patients, all of whom were manual laborers. Sixteen of them returned to full duties in an average of 5.6 months (range: 4.7-6.6 months). Four patients had their jobs modified to light-duty roles, whereas two patients had not returned to work during the follow-up period (eight months for one, and 8.5 months for the other). Three patients (5.6%) had reoperation for implant removal because one or more screws had become prominent medially and symptomatic. One patient had a screw removed on the 35th postoperative day because one of the screws was penetrating the subtalar joint as assessed during the postoperative CT scan.

Range of motion was assessed postoperatively and it displayed mean dorsiflexion of 20.3 degrees (range: 5-26), mean plantar flexion of 29 degrees (range: 11-50), mean subtalar inversion of 15.5 degrees (range: 10-31), and mean eversion of 5.8 degrees (range: 3-10) [18]. Bohler’s angle, Gissane angle, and posterior facet joint depression were assessed in all eligible patients preoperatively, immediately postoperatively, and delayed postoperatively after initiating weight bearing. Statistically significant improvements were observed in the restoration of the posterior facet joint depression and Bohler’s angle (p-value < 0.005) in the postoperative radiographs.

## Discussion

Operative versus non-operative treatment for calcaneus fractures has been debated for a long time. With conservative treatment, the fracture fragments usually heal together, but the calcaneus remains deformed, the joint surfaces are incongruous, and the alignment of the leg through the ankle to the heel is lost. Severe, painful osteoarthritis of the subtalar joint often follows. Recovery is prolonged and typically takes about two years. Even then, most patients end up having a painful, stiff, deformed foot and are unable to wear a normal shoe; walking is painful and many need the assistance of a walking stick. These poor outcomes are especially problematic for manual laborers and outdoor workers as they are unable to resume their occupations. This effect on work-life was recognized as early as 1916: “Ordinarily speaking, the man who breaks his heel bone is done, so far as his industrial future is concerned [19].” Howard and colleagues [6] found that significant complications following intra-articular fractures occurred whether treated operatively or non-operatively, despite management by experienced surgeons [4,5]. Soft tissue complications, such as compartment syndrome, fracture blisters, full-thickness skin necrosis, and peroneal tendon pathology, can be also seen in patients treated non-operatively, illustrating the point that even conservative management can result in suboptimal outcomes. Although operative treatment is not superior in managing displaced intra-articular calcaneus fractures at one year of follow-up, it appears to have greater benefits at eight to 12 years postoperatively. Operative treatment was associated with a higher risk of complications but also enjoyed reduced post-traumatic arthritis on follow-up radiographs [8,16]. Moreover, operative treatment results in improved functional outcomes and better restoration of radiographic parameters [20,21].

The most commonly used approach for operative fixation of calcaneal fractures is the extensile lateral approach in a lateral decubitus position popularized by Benirschke and Sangeorzan [9,11]. In our study, we have described this approach in a distracted prone position which we believe is beneficial in achieving most goals of the open reduction internal fixation, which are mainly 1. restoration of the height, 2. correction of the varus deformity, 3. correction of the medial displacement, and 4. minimal soft tissue handling.

By virtue of distraction gained from the overhead hanging of the injured lower limb by a Steinmann pin through the calcaneus tuberosity, we were able to counteract the deforming forces by the Achilles tendon. The position helped in gaining almost spontaneous restoration of height, correction of varus deformity, easier correction of the medial displacement with minimal manipulation of the fracture pieces, and minimal handling of the soft tissue flap that will tilt away from the surgical field by virtue of gravity.

In our series, we encountered a low rate of wound complications, and only one case required surgical intervention. The rate of serious infection and wound necrosis complications following open treatment of closed fractures in most reported series is approximately 2%-27% [12-14]. Figures reported by the Folk study [10] have shown that after a standard extensile L-shaped approach with two-layer flap closure, wound complications developed in 25% of patients, with 21% requiring further surgery for these complications. A prospective and randomized study by Griffin et al. [7] demonstrated that complication rates were much higher in operative treatment, with a 19% infection rate and 11% requiring secondary surgery to remove infected or painful screws and plates. We believe this low wound complication rate is due to the minimal fracture piece manipulation and soft tissue handling gained by the distracted prone position.

The reoperation rate in our series is 5.6%, which is also low compared with previous studies that showed 19.2% and 50% reoperation rates by minimally invasive approach and classic extensile group, respectively [20]. More than 50% of the patients who were operated on with this technique were able to obtain a good postoperative range of motion comparable to normal values [18].

With an average period of return to full duties at 5.6 months (range: 4.7-6.6 months) for most of our patients, who are manual laborers, we believe that we were able to achieve our goal successfully in these patients. Four patients had their jobs modified to light-duty roles, and two patients had not returned to work during the follow-up period (eight months for one, and 8.5 months for the other).

There are certain limitations to the current study. We do understand that there is a loss in long-term follow-up, but most of our patients were expatriates and many had returned to their respective home countries during the follow-up period. Although there was no radiographic evidence of subtalar evidence in the postoperative visits, we understand that a longer follow-up may be warranted for a better determination. The study does not include a control group as the main surgeon operated on all the calcaneus fractures with the same technique. A larger study with a control group can be done in the future for comparative results. The majority of our patients were laborers and relatively healthy individuals, which may suggest poor generalizability, but this can be attributed to the fact that most patients in our region sustaining this type of injury belong to the same cohort. All the cases were operated on by a single senior surgeon, which can be a reason for surgeon-specific confounders, but no other surgeon in the institute was employing this approach. Moreover, we hope that a larger study involving multiple surgeons can be undertaken in the future to avoid these limitations.

## Conclusions

In conclusion, operative treatment of calcaneus fractures by open reduction and internal fixation in a distracted prone position has shown a low rate of the wound and soft tissue complications with good radiographic and clinical results in our series. To our knowledge, this technique has not been published in the literature before. Despite its limitations, we believe that it is an effective alternative technique for approaching calcaneus fractures. As indicated in our results, this specific approach can be used for patients with calcaneus fractures to obtain desired outcomes. We anticipate that our technique will prove useful to surgeons in their practice.
